# A Combined Phenotypic-Genotypic Predictive Algorithm for In Vitro Detection of Bicarbonate: β-Lactam Sensitization among Methicillin-Resistant *Staphylococcus aureus* (MRSA)

**DOI:** 10.3390/antibiotics10091089

**Published:** 2021-09-09

**Authors:** Selvi C. Ersoy, Warren E. Rose, Robin Patel, Richard A. Proctor, Henry F. Chambers, Ewan M. Harrison, Youngju Pak, Arnold S. Bayer

**Affiliations:** 1 The Lundquist Institute, Torrance, CA 90502, USA; selvi.ersoy@lundquist.org (S.C.E.); ypak@lundquist.org (Y.P.); 2 School of Pharmacy, University of Wisconsin-Madison, Madison, WI 53705, USA; warren.rose@wisc.edu; 3 Mayo Clinic, Rochester, MN 55902, USA; patel.robin@mayo.edu; 4 School of Medicine and Public Health, University of Wisconsin, Madison, WI 53706-152, USA; rap@wisc.edu; 5 School of Medicine, University of California San Francisco, San Francisco, CA 94143, USA; Henry.Chambers@ucsf.edu; 6 Wellcome Sanger Institute, Hinxton CB10 1SA, UK; eh6@sanger.ac.uk; 7 Department of Medicine, University of Cambridge, Cambridge CB2 0QQ, UK; 8 Department of Public Health and Primary Care, University of Cambridge, Cambridge CB2 0SR, UK; 9 Geffen School of Medicine, University of California Los Angeles, Los Angeles, CA 90025, USA

**Keywords:** methicillin-resistant *Staphylococcus aureus* (MRSA), β-lactam susceptibility, sodium bicarbonate (NaHCO_3_), antimicrobial susceptibility testing (AST), genome sequencing

## Abstract

Antimicrobial susceptibility testing (AST) is routinely used to establish predictive antibiotic resistance metrics to guide the treatment of bacterial pathogens. Recently, a novel phenotype termed “bicarbonate (NaHCO_3_)-responsiveness” was identified in a relatively high frequency of clinical MRSA strains, wherein isolates demonstrate in vitro “susceptibility” to standard β-lactams (oxacillin [OXA]; cefazolin [CFZ]) in the presence of NaHCO_3_, and in vivo susceptibility to these β-lactams in experimental endocarditis models. We investigated whether a targeted phenotypic-genotypic screening of MRSA could rule in or rule out NaHCO_3_ susceptibility upfront. We studied 30 well-characterized clinical MRSA bloodstream isolates, including 15 MIC-susceptible to CFZ and OXA in NaHCO_3_-supplemented Mueller–Hinton Broth (MHB); and 15 MIC-resistant to both β-lactams in this media. Using a two-tiered strategy, isolates were first screened by standard disk diffusion for susceptibility to a combination of amoxicillin-clavulanate [AMC]. Isolates then underwent genomic sequence typing: MLST (clonal complex [CC]); *agr*; SCC*mec*; and *mecA* promoter and coding region. The combination of AMC disk susceptibility testing plus *mecA* and *spa* genotyping was able to predict MRSA strains that were more or less likely to be NaHCO_3_-responsive in vitro, with a high degree of sensitivity and specificity. Validation of this screening algorithm was performed in six strains from the overall cohort using an ex vivo model of endocarditis. This ex vivo model recapitulated the in vitro predictions of NaHCO_3_-responsiveness vs. nonresponsiveness above in five of the six strains.

## 1. Introduction

Antibiotic resistance is a major threat to healthcare initiatives worldwide [1,2,3]. Despite the availability of extensive in vitro testing methods and international consensus committee interpretive guidelines to aid in treatment practices, antibiotic resistance has not abated [3,4,5,6]. Of note, there has been a lack of innovation in the development of in vitro methods that mimic the host microenvironment and milieu during active infection [7,8,9].

Guideline committees, such as the Clinical and Laboratory Standards Institute (CLSI) and the European Committee on Antimicrobial Susceptibility Testing (EUCAST) generally rely heavily on “standard” in vitro methods of antimicrobial susceptibility testing (AST) to determine minimum inhibitory concentrations (MICs) of antibiotics towards various pathogens. These standardized methods utilize specific growth media, such as Mueller–Hinton Broth/Agar (MHB/A), for most bacterial pathogens [4,10]. For select antibiotics, specific supplements are included or depleted to ensure appropriate expression of resistance genes or prevent degradation of antibiotics in an artificial medium [11,12,13]. However, this medium, even with alterations, is not necessarily reflective of host environmental conditions pathogens may face during infection [7]. Primary AST methods employed in clinical microbiology laboratories include broth microdilution (BMD), agar dilution, and/or disk or gradient diffusion strip assays [6,14,15]. Manual BMD and agar dilution MICs are time- and labor-intensive and not widely used [6,16]. Advancements have been made to machine-automate this process (e.g., the Becton–Dickinson Phoenix or the BioMérieux Vitek 2 systems [17,18]). Increased ease and cost-effectiveness of bacterial whole genome sequencing (WGS) may allow clinical microbiology laboratories to incorporate genomic identification of specific resistance genes and mutations as an accompaniment to routine in vitro screening methods in the near future [17,19,20,21,22]. To date, only a relatively small number of laboratories have adopted routine WGS, mostly for molecular epidemiology and infection control purposes.

Recent studies have highlighted potential limitations of relying on standard methods of AST to determine relevant antibiotic resistance in specific infection settings [8,9,23,24,25,26]. These studies utilized media that may be more reflective of the host environment, such as tissue culture media (e.g., RPMI) or MHB supplemented with NaHCO_3_, the body’s primary biological buffer, to perform in vitro AST. Results of such studies suggest that it could be possible that “antibiotic resistance levels by MICs” determined in host-like media are better predictors of host infection outcomes in both murine and rabbit systemic infection models [8,9,23,24,25]. Our laboratory has recently published a series of investigations documenting the following in terms of MIC testing of MRSA in NaHCO_3_-supplemented MHB: (i) ~33% and ~75% frequency of in vitro susceptibility of MRSA to OXA and CFZ, respectively; (ii) better clearance of in vitro NaHCO_3_-responsive MRSA from experimental cardiac vegetations in both ex vivo and in vivo models; and (iii) definition of important mechanisms involved in this NaHCO_3_-responsive phenotype (e.g., penicillin-binding protein 2a (PBP2a) production, translocation and maturation) [23,26,27,28]. 

The aim of this exploratory and hypothesis-generating study was to combine current AST methods with targeted genetic sequencing to identify MRSA that may potentially respond to standard β-lactam therapy in vivo [23,26].

## 2. Results

### 2.1. Identification of Phenotypic and Genotypic Traits Associated with NaHCO_3_-Responsiveness

Within this 30-isolate collection (summarized in Table S1), two *mecA* genotypes were detected, termed “susceptible 2” and “resistant 2”, as per Harrison et al. [29]. The susceptible 2 genotype has a T at the *mecA* promoter -7 ribosomal-binding site (RBS), whereas the resistant 2 genotype has a G at this site. This promoter single nucleotide polymorphism (SNP) is associated with decreased *mecA* transcription in susceptible 2 MRSA as compared to resistant 2 isolates [29]. Both Chi-squared and Fisher’s exact analysis revealed that the susceptible 2 genotype were significantly associated with a NaHCO_3_-responsive phenotype (Table S2, *p* < 0.01). However, the linkage between the responsiveness phenotype and the susceptible 2 genotype was highly sensitive (93.3%, 95% CI [68.1–99.8]), the use of this single metric as a predictor of responsiveness resulted in a specificity of only 66.7% (95% CI [38.4–88.2]).

We investigated the correlation between NaHCO_3_-responsiveness and susceptibility to amoxicillin + clavulanate (AMC) by standard disk diffusion assay. AMC disks were employed due to their commercial availability (in comparison to penicillin–clavulanate combination disks). The 2012 CLSI-defined staphylococcal breakpoints for AMC by disk diffusion are given as S ≥ 20 mm and R ≤ 19 mm [30]. Of note, these CLSI parameters were ultimately replaced by generally reporting ‘OXA susceptibility’ as a surrogate for presumed susceptibility to such β-lactam-β-lactamase inhibitor combinations and have not been updated since 2013 [31,32,33]. Interestingly, despite the published association between the *mecA* susceptible 2 genotypes and susceptibility to penicillin + CA by E-test [29], we found no significant correlations between this genotype and susceptibility to AMC by disk diffusion, using the above breakpoints (Table 1). 

For the present study, to try to improve the utility of AMC disk diffusion results in screening for NaHCO_3_-responsive MRSA, we defined a distinct set of breakpoints (S ≥ 15 mm and R ≤ 14 mm) based on the overall zone size median among the overall isolate-set, as well as the zone size mode among the NaHCO_3_-responsive cohort. Applying this revised metric to our 30 MRSA strains, “susceptibility” as defined by AMC disk diffusion profiles correctly identified 12/15 NaHCO_3_-responsive isolates (sensitivity of 80.0% (95% CI [51.9–95.7]). However, the specificity of this metric was only 33.3% (95% CI [11.8–61.6%]) (Table S2). Furthermore, the receiver operator curve (ROC) analysis revealed the AUC of AMC susceptibility testing as a predictor of NaHCO_3_-responsiveness to be less than 0.5 (data not shown), indicative of a poor diagnostic test.

To improve the specificity of the AMC disk diffusion assay for predicting the NaHCO_3_-responsive phenotype, we combined this latter assay with targeted genotypic profiling (i.e., MLST [CC], *spa*, *agr*, SCC*mec* and *mecA* coding-promoter genotypes) (Table 1 and Table S2). These analyses revealed that NaHCO_3_-responsive isolates could be predicted by their susceptibility to AMC in combination with the presence of the *mecA* “susceptible 2” genotype, plus either *spa* type t008 or t002 (Table 2 and Table S2). Although this combinatorial method had a sensitivity of only 66.7% (95% CI 38.4–88.2%), it exhibited 100% specificity with a 95% CI of 78.2–100%. Thus, the initial screening of strains by AMC disk diffusion, if ‘resistant’ by the above-customized breakpoints, would potentially eliminate a need for subsequent genotypic analysis of MRSA strains. 

### 2.2. Ex Vivo Validation of NaHCO_3_-Responsiveness Screening Criteria

The NaHCO_3_-responsive strains chosen for ex vivo validations (as defined by BMD testing NaHCO_3_-supplemented MHB; C24, C32, C38) met all three of the above in vitro screening metrics (Table 1). In contrast, although all the nonresponsive strains (as defined by BMD testing; RB 057-171, C15, PB 321-236) displayed susceptibility to AMC by disk diffusion, only one strain had a susceptible *mecA* genotype, and none displayed a t008 or t002 *spa* type (Table 1). 

**Figure 1:**
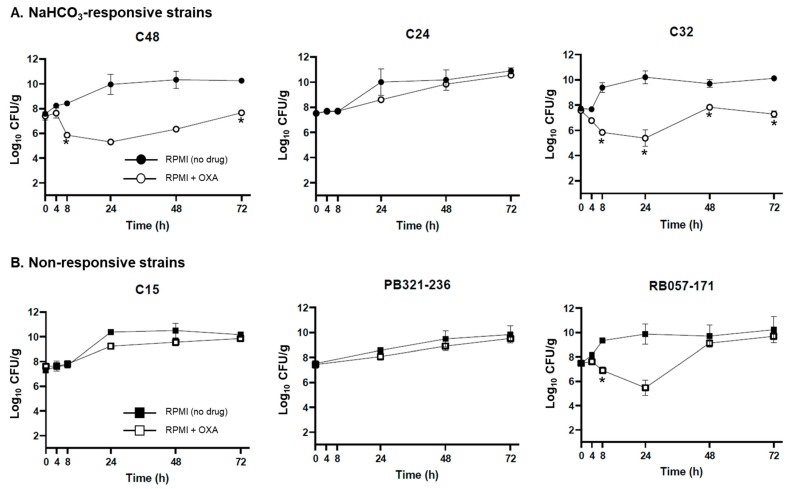
Simulated endocarditis vegetation (SEV) tissue burdens following OXA treatment of (**A**) NaHCO_3_-responsive or (**B**) Nonresponsive MRSA. SEVs containing MRSA were exposed to RPMI tissue culture medium with or without OXA exposure at doses that simulate human serum PK/PD. Vegetations were quantitatively cultured for MRSA CFU/g at 0, 4, 8, 24, 48, and 72 h time points. Statistics were performed on the CFU/g present in untreated samples versus OXA treatment at the indicated time point by a Student’s *t*-test (* *p* < 0.05).

In the ex vivo model, five of the six validation strains recapitulated the responsiveness phenotypes, as well as the algorithm predictions proposed in vitro. Thus, responsive strains C48 and C32 demonstrated significant reductions in MRSA counts by OXA over the 72 h exposures in the SEV model, especially early (24 h); however, growth of the C24 strain was not inhibited by OXA in this model (Figure 1A). None of the nonresponsive strains were inhibited by OXA by the 72 h time point (Figure 1B), although strain RB 057-171 demonstrated early OXA-killing (24 h).

## 3. Discussion

Given the complexity, costs and treatment-related toxicities of current standard-of-care therapies for invasive MRSA infections (e.g., vancomycin, daptomycin, linezolid) [34,35,36], the use of effective, inexpensive and well-tolerated antibiotics (such as β-lactams) would be advantageous. However, current clinical guidelines discourage the use of β-lactams in MRSA infections because of their uniform “in vitro resistance” to such agents by standard MIC testing [15,32]. As noted above, we have previously identified that a significant proportion of clinical MRSA isolates exhibit susceptibility in vitro to standard β-lactams if tested in the presence of NaHCO_3_ [26]. However, routine AST of all MRSA isolates in both standard and NaHCO_3_-supplemented media by BMD would involve additional reagent and materials costs, technical effort, as well as turnaround time implications for clinical microbiology laboratories.

**Figure 2:**
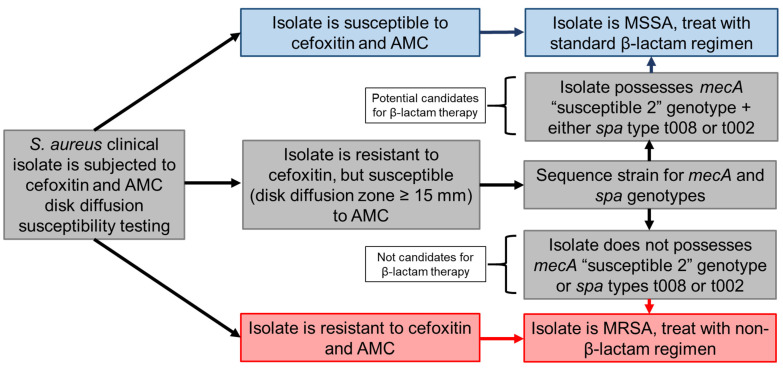
Algorithm for determining MRSA strains which are potentially “β-lactam-susceptible”. Isolates initially determined to be *S. aureus* are subjected to standard disk diffusion testing (cefoxitin; AMC). Those strains which are resistant to cefoxitin but ‘susceptible’ to AMC, based on a modified breakpoint zone size cut off of ≥15 mm, then undergo targeted genotyping. Only those such isolates which possess both the *mecA* “susceptible 2” plus either *spa* type t008 or t002 genotypes would then be considered as potentially “in vivo susceptible” to β-lactams.

In the present study, we attempted to establish a specific algorithm for the identification of NaHCO_3_-responsive MRSA utilizing both standard methods currently employed by clinical microbiology laboratories, as well as genotypic screening modes. This is a preliminary and exploratory study designed to generate metrics for specifically identifying NaHCO_3_-responsive strains in vitro that will likely respond to β-lactam therapy in vivo. Our proposed algorithm (summarized in Figure 2) relies on initial screening of MRSA by a standard disk diffusion assay using AMC, employing a susceptibility breakpoint of ≥15 mm zone sizes. Following this initial phenotypic ‘checkpoint’, isolates that meet this criterion (i.e., AMC-‘susceptible’) would then undergo targeted genomic sequencing to specifically determine their *mecA* coding and RBS sequences, and *spa* genotypes to identify strains as potential candidates for β-lactam therapy. As WGS becomes more frequently used in clinical laboratories, these provisional genotypic criteria may be further refined as screening metrics to predict NaHCO_3_-responsiveness with a higher degree of sensitivity and specificity. This targeted genotyping profiling presented here differs from currently employed strategies for WGS-based AST, wherein isolates are screened for specific genes/alleles with known mechanistic associations with well-defined antibiotic resistance mechanisms [17,20]. In contrast, our current data suggest that the specific mechanisms involved in the β-lactam-NaHCO_3_-responsiveness phenotype are multifactorial, and not related to perturbations of a single gene locus [23,28]. Thus, the screening method described here relies on the association of certain genotypic-phylogenetic markers with the β-lactam-NaHCO_3_-responsiveness phenotype, although these genotypes may not directly underlie this phenotype.

The screening algorithm above is highly specific; thus, strains that do not fulfill all three phenotypic-genotypic metrics are unlikely to be β-lactam-NaHCO_3_-responsive. However, this algorithm lacks the sensitivity inherent in direct AST of MRSA isolates in NaHCO_3_-containing media by BMD MIC. Despite this lack of sensitivity, this proposed screening algorithm underscores the ability to classify MRSA as unlikely candidates for β-lactam therapy, and is a highlight of this approach.

Manual BMD AST is both time- and labor-intensive, and therefore, many clinical laboratories either automate the process using commercial systems or disk diffusion testing when CLSI interpretive criteria are available [37,38]. Theoretically, alternative media (e.g., NaHCO_3_-supplemented) could be incorporated into commercially-available automated systems, although such changes would require extensive diagnostic validations and quality controls by manufacturers. However, if clinical trial data demonstrate improvements in patient outcomes associated with the use of a β-lactam over alternative therapies, this change in diagnostics might well be driven by clinical need.

Our previous larger screening of a 58 isolate MRSA collection, from which the 30 isolate subset used in this study was derived, revealed a statistical linkage between the presence of CC8 and *spa* t008 genotypes and the NaHCO_3_-responsive phenotype [26]. This indicated that many isolates exhibiting the NaHCO_3_-responsive phenotype might have arisen from a specific clonal lineage. However, this link was not strong enough to utilize these genotypes as the sole method to identify NaHCO_3_-responsive MRSA. Formal phylogenetic and bioinformatic analyses of a larger number of NaHCO_3_-responsive/nonresponsive MRSA will be required to provide further, more definitive, insights into the ancestral origins of this phenotype and point to additional genetic sequence targets to aid in the rapid genomic identification of such strains. 

The *mecA* gene locus encodes the alternative penicillin-binding protein PBP2a, which is considered to be the primary determinant of β-lactam resistance in MRSA [39,40]. Many clinical laboratories routinely screen for the presence of *mecA*. However, intra-locus SNP analysis (as done in this study) is not generally employed to subcategorize *mecA* genotypes; such analytics appear to be informative, given the importance of specific *mecA* coding sequence-promotor genotypes in determining susceptibility to certain β-lactam-β-lactamase inhibitor combinations [29]. Of interest, we determined that certain “susceptible” *mecA* genotypes were significantly associated with the NaHCO_3_-responsive phenotype. However, screening for specific *mecA* genotypes alone did not appear to be highly specific (66.7%, CI [38.4–88.2%]), and could result in many MRSA isolates being erroneously identified as NaHCO_3_-responsive as a single determinant.

Most of our six validation strains exhibited the expected outcomes as predicted by the algorithm in the ex vivo SEV model. Interestingly, the nonresponsive strain RB 057-171 (as determined by in vitro testing in NaHCO_3_-containing media), displayed an initial reduction in bacterial vegetation burden following OXA treatment at 24 h. This strain displayed susceptibility to AMC by disk diffusion and also possessed the *mecA* “susceptible 2” genotype but did not possess a *spa* type of t008 or t002. This outcome indicates that strains possessing the *mecA* “susceptible 2” genotype may demonstrate some short-term β-lactam susceptibility in the SEV model, which abates over a longer exposure time-course. This finding further emphasizes the need for additional screening metrics (i.e., *spa* typing) to ‘rule out’ strains as candidates for clinical β-lactam treatment. The in vitro responsive strain, C24, was not inhibited by OXA in the SEV model, despite meeting all three algorithmic screening criteria. This underscores the need for further validation testing of a larger collection of strains in both ex vivo and in vivo models.

The discovery of bacterial antimicrobial susceptibility profiles as they occur during host infection and determining genetic signatures associated with these phenotypes can usher in a new era of precision antibiotic treatment practices. As WGS and other genotyping methods become faster, cheaper, and more widespread, the identification of specific sequences and genomic markers associated with antibiotic-resistant and antibiotic-susceptible phenotypes will undoubtedly be employed by many clinical microbiology laboratories. Today, WGS is considered the state-of-the-art method for clonality testing of *S. aureus*, having replaced outdated methods such as pulsed-field gel electrophoresis [41,42,43,44].

Turnaround times of data to clinicians caring for the patients in real-time, as well as microbiology laboratory costs (both technical and reagents), are important considerations for the implementation of novel AST screening methods. Our current best estimates are that the combination of AMC disk diffusion plus *spa* typing/*mecA* sequencing is estimated to take ~3 days. In contrast, BMD testing of strains in parallel in both standard media, as well as onsite preparation of NaHCO_3_-supplemented media, is estimated to take up to 4 days. Further, CA-MHB-NaHCO_3_ BMD testing would certainly be more laborious for the laboratory than AMC disk diffusion testing. Finally, bacterial whole genome sequencing (WGS) and annotation (if all equipment is onsite) are predicted to take ~2–3 days. These estimates are dependent on the availability of adequately trained laboratory staff seven days a week, daily batching of testing, and pre-preparation of media/reagents [45]. 

It is difficult to put a comparative and accurate cost-out for each of the above three potential screening methodologies. However, it is generally agreed that sequencing, whether using next-generation sequencing, targeted sequencing of one or more genes of interest (e.g., using Sanger sequencing) is more expensive than disk diffusion or MIC testing; this includes both cost of sequencing preparation, sequencing itself, as well as for analytics. 

Our study has certain distinct limitations, including: (i) a relatively small sample size of β-lactam-NaHCO_3_-responsive and -nonresponsive MRSA; this predictive algorithm needs to be applied to a significantly larger cohort of isolates to further examine its sensitivity and specificity; current work is being undertaken to screen a much larger collection of skin and soft tissue MRSA isolates (*n* = 105) to identify further NaHCO_3_-responsive isolates to aid in the validation of this algorithm; (ii) lack of geographic diversity of the study strains (US only); multi-national strain cohorts need to be evaluated with this algorithm; (iii) focus only upon bloodstream isolates; additional study of isolates from other common infection syndromes (e.g. skin and soft tissue infections) needs to be carried out; and (iv) limited scope of validation of this in vitro predictive algorithm in translational ex vivo and in vivo models of invasive MRSA infections treated with selected β-lactams; current work is being undertaken to validate NaHCO_3_-responsive and nonresponsive strains in a rabbit model of infective endocarditis (IE).

## 4. Materials and Methods

### 4.1. Bacterial Strains, Growth Conditions, and Susceptibility Testing

A collection of 15 NaHCO_3_-responsive and 15 NaHCO_3_-nonresponsive isolates was used in this study. All of these isolates have been previously characterized for their susceptibility to OXA and CFZ in the presence or absence of NaHCO_3_ [23,26,28,29]. This 30-isolate cohort represents clonal complex types common in worldwide circulation, as well as a diversity of baseline OXA and CFZ MICs (as determined in CA-MHB) (Table S1). All isolates were stored at −80 °C until thawed for use and cultured on Tryptic Soy Agar (TSA). For MIC testing, we utilized BMD; strains were cultured in cation-adjusted Mueller Hinton Broth (CA-MHB) ± 100 mM Tris buffer (to maintain a stable pH) ± 44 mM NaHCO_3_ (pH 7.3 ± 0.1). For MIC testing in OXA, 2% NaCl was also included in the MHB. Although 44 mM of NaHCO_3_ is above standard human blood levels, it reflects tissue levels [46]. In addition, MIC determinations among MRSA using this latter NaHCO_3_ level (vs. 25 mM) correlates better with ex vivo and in vivo infection model outcomes [23,27]. For MIC testing, isolates were grown overnight, then diluted into the specific AST testing media-of-interest, as previously described [23]).

Harrison et al. [29] have previously shown among selected MRSA isolates, there is a significant relationship between i) specific *mecA* coding region and RBS promoter genotypes; and ii) in vitro E-test susceptibility to combinations of β-lactams (e.g., penicillin) with clavulanic acid (CA). In the current study, we employed commercially available AMC disks in a Kirby–Bauer diffusion assay on MHA using a standard inoculum [47]. Based on the overall distribution of zone diameters among the current 30 isolate collection, as well as mean and modal zone sizes, we used customized breakpoints of “susceptible” ≥ 15 mm and “resistant” ≤ 14 mm. Assays were done at least in duplicate on separate days, with zone size readings done blinded as to the β-lactam-NaHCO_3_-responsiveness phenotype.

### 4.2. mecA, Clonal Complex (CC), agr, SCCmec, and spa Genotyping

To identify specific polymorphisms within the *mecA* promoter and coding regions, DNA was isolated from MRSA strains by chelation (InstaGene-Matrix, Bio-Rad, Hercules, CA, USA) and the *mecA* region amplified by PCR using Phusion High-Fidelity DNA Polymerase (New England Biolaboratories) (amplification and sequencing primers listed in Table S3). PCR products were purified using the PureLink PCR Micro Kit (Invitrogen, Waltham, MA, USA) and sequenced by GeneWiz LLC. 

For determination of CC, SCC*mec*, *agr*, and *spa* genotypes, *S. aureus* DNA was isolated by boiling, followed by treatment with 20 µg/mL lysostaphin (Sigma-Aldrich, Merck KGaA, Darmstadt, Germany); the lysate was then used as a template for PCR. *spa* typing was performed as described [48], and the resulting repeat patterns were used to infer MLST–based CC types [49]. SCC*mec* typing was performed using a molecular beacon-based real-time PCR assay [50]. Finally, *agr* typing was performed using a modified version multiplex PCR assay [51]. Results of these four latter genotype profiles have been previously reported [26].

### 4.3. Statistical Analyses

Associations between various phenotypic and genotypic metrics and the NaHCO_3_-responsive vs. nonresponsive phenotypes were summarized in 2 × 2 cross-tabulation tables, and analyzed by two-tailed Chi-square analytics and Fisher’s exact test where appropriate. A Student’s *t*-test was used for comparisons of MRSA bioburdens within the SEV model for each isolate. Determinations of sensitivity and specificity and their statistical relevance, for a given set of criteria, were calculated by MedCalc statistical software (https://www.medcalc.org/calc/diagnostic_test.php, accessed date: 13 January 2021). ROC analysis was performed using a web-based calculator (http://www.rad.jhmi.edu/jeng/javarad/roc/JROCFITi.html, accessed date: 12 February 2021).

### 4.4. Pharmacodynamic Model with Ex Vivo Simulated Endocardial Vegetations (SEVs)

This ex vivo model was performed as described previously [27,52], to attempt to recapitulate the in vitro designations of NaHCO_3_-responsive vs. nonresponsive phenotypes (as predicted using the three-metric algorithm of AMC susceptibility, plus *mecA* and *spa* genotyping). Three isolates fulfilling all three metrics were randomly selected and compared ex vivo to three isolates not fulfilling these three metrics. SEVs consisted of pooled human cryoprecipitate antihemophilic factor prepared from plasma (fibrinogen, von Willebrand factor, factor VIII, factor XIII and fibronectin) and pooled platelets were collected from human volunteer donors (UW Health Blood Bank, Madison, WI, USA). Bovine thrombin (UW Health, Madison, WI, USA) and aprotinin (Sigma-Aldrich) were commercially purchased. SEVs contain 3–3.5 g/dL of albumin and 6.8–7.4 g/dL of total protein (equating to human physiologic levels) [53,54]. 

In the central fluid phase compartment, a 150 mL flask was prefilled with 100 mL RPMI media, which contains 25 mM NaHCO_3_ and magnetic stir bar, and the SEVs were added for 30 min prior to antibiotic dosing, to allow for climate acclimation. OXA (2 g) was infused every 4 h (over 5 min) using a simulation derived from human PK/PD data (C_min_ 2.3 µg/mL, half-life 1.0 h, k_e_ 0.69/h) and standard dosing regimens for patients with severe MRSA infections [55,56]. Samples of SEVs were taken at specified time points, weighed, digested, and plated for MRSA quantification (CFU/g of SEV tissue).

## Figures and Tables

**Table 1:** Disk diffusion susceptibility to β-lactam/β-lactamase inhibitors and genotypes for various loci in NaHCO_3_-responsive and nonresponsive MRSA strains.

Responsive Strains (*n* = 15)							
Strain	AMC ^A^	*mecA* Genotype	Ridom *spa* Type	*agr* Type	CC Type	SCC*mec* Type	β-lactamase (±)
MRSA 11/11	16 (S)	susceptible 2	t008	*agr* I	8	IV	+
MW2	20 (S)	susceptible 2	t128	*agr* III	1	IV	+
BCVA289	16 (S)	susceptible 2	t008	*agr* I	8	IV	+
PB 031-038	11 (R)	resistant 2	Unknown ^B^	*agr* I	8	IV	+
PB 004-193	15 (S)	susceptible 2	t008	*agr* I	8	IV	+
PB 043-043	15 (S)	susceptible 2	t008	*agr* I	8	IV	+
PB 077-107	13 (R)	susceptible 2	t002	*agr* II	5	II	+
C48	18 (S)	susceptible 2	t008	*agr* I	8	IV	+
C42	15 (S)	susceptible 2	t008	*agr* I	8	IV	+
C13	15 (S)	susceptible 2	t008	*agr* I	8	IV	+
C32	18 (S)	susceptible 2	t002	*agr* II	5	IV	+
C30	19 (S)	susceptible 2	t008	*agr* I	8	IV	+
C24	16 (S)	susceptible 2	t008	*agr* I	8	IV	+
C38	14 (R)	susceptible 2	t008	*agr* I	8	IV	+
RB 300-087	18 (S)	susceptible 2	t2265	*agr* I	45	IV	+
**Nonresponsive Strains (*n* = 15)**							
**Strain**	**AMC**	***mecA* Genotype**	**Ridom *spa* type**	***agr* type**	**CC type**	**SCC*mec* type**	**β-lactamase (±)**
BMC1001	15 (S)	resistant 2	t064	*agr* I	8	IV	+
C5	26 (S)	resistant 2	t242	*agr* II	5	II	+
RB 067-227	18 (S)	susceptible 2	t128	*agr* III	1	IV	+
RB 010-016	11 (R)	resistant 2	t002	*agr* II	5	II	+
PB 027-133	13 (R)	resistant 2	t002	*agr* II	5	II	+
PB 088-180	23 (S)	resistant 2	t002	*agr* II	5	II	+
RB 034-221	23 (S)	resistant 2	t002	*agr* II	5	II	+
C7	14 (R)	susceptible 2	t008	*agr* I	8	IV	+
C36	14 (R)	resistant 2	t002	*agr* II	5	II	+
C15	16 (S)	resistant 2	t064	*agr* I	8	IV	+
PB 300-111	17 (S)	susceptible 2	t051	*agr* I	8	IV	+
PB 321-236	23 (S)	resistant 2	t003	*agr* II	5	II	+
C3	14 (R)	susceptible 2	t008	*agr* I	8	IV	+
PB 017-037	24 (S)	resistant 2	t002	*agr* II	5	II	+
RB 057-171	15 (S)	susceptible 2	t9878	*agr* III	1	IV	+

^A^ Zone diameter (mm) for amoxicillin + clavulanate (AMC) disk diffusion assay, classified as Resistant = (R) or Susceptible = (S) based on a newly defined breakpoint of R ≤ 14 mm and S ≥ 15 mm; ^B^ Unknown repeat succession: r11r19r12r05r25.

**Table 2:** Selected screening criteria for identifying NaHCO_3_-responsive and nonresponsive MRSA. Criteria are AMC susceptible, *mecA* “susceptible 2” genotype, *spa* type t008 or t002. Only strains meeting all three of these criteria are considered NaHCO_3_-responsive (Chi-squared and Fisher’s exact *p* < 0.01).

Algorithm Criteria Met?	NaHCO_3_-Responsive	Nonresponsive
Criteria met	10	0
Criteria not met	5	15
**Statistic**	**Value**	**95% CI**
Sensitivity	66.67%	38.38% to 88.18%
Specificity	100.00%	78.20% to 100.00%

## Data Availability

The data presented in this study are available in Table S1 of this article.

## Supplementary Materials

The following are available online at https://www.mdpi.com/article/10.3390/antibiotics10091089/s1, Table S1: Phenotypic and genotypic characterization of NaHCO_3_-responsive and nonresponsive MRSA strains, Table S2: Summary of statistical analyses of various algorithms for identifying NaHCO_3_-responsive MRSA, Table S3: *mecA* sequencing primers.

## References

[1] Centers for Disease Control and Prevention (2013). Antibiotic resistance threats in the United States, 2013. Threat Report.

[2] World Health Organization (2014). Antimicrobial Resistance: Global Report on Surveillance.

[3] Abadi A.T.B., Rizvanov A.A., Haertlé T., Blatt N.L. (2019). World Health Organization report: Current crisis of antibiotic resistance. BioNanoScience.

[4] World Health Organization (1961). Standardization of Methods for Conducting Microbic Sensitivity Tests-Second Report of the Expert Committee on Antibiotics.

[5] Weinstein M.P., Patel J.B., Campeau S., Eliopoulos G.M., Galas M.F., Humphries R.M., Jenkins S.G., Limbago B., Mathers A.J., Mazzulli T. (2018). Performance Standards for Antimicrobial Susceptibility Testing.

[6] Tibbetts R.J. (2018). Antimicrobial Susceptibility Testing Paradigms: Current Status and Future Directions. Am. Soc. Clin. Lab. Sci..

[7] Nizet V. (2017). The accidental orthodoxy of Drs. Mueller and Hinton. EBioMedicine.

[8] Kubicek-Sutherland J.Z., Heithoff D.M., Ersoy S.C., Shimp W.R., House J.K., Marth J.D., Smith J.W., Mahan M.J. (2015). Host-dependent induction of transient antibiotic resistance: A prelude to treatment failure. EBioMedicine.

[9] Ersoy S.C., Heithoff D.M., Barnes L.T., Tripp G.K., House J.K., Marth J.D., Smith J.W., Mahan M.J. (2017). Correcting a fundamental flaw in the paradigm for antimicrobial susceptibility testing. EBioMedicine.

[10] Cockerill F.R. (2012). Methods for Dilution Antimicrobial Susceptibility Tests for Bacteria That Grow Aerobically: Approved Standard.

[11] Chambers H.F., Hackbarth C.J. (1987). Effect of NaCl and nafcillin on penicillin-binding protein 2a and heterogeneous expression of methicillin resistance in *Staphylococcus aureus*. Antimicrob. Agents Chemother..

[12] Andrews J.M., Baquero F., Beltran J.M., Canton E., Crokaert F., Gobernado M., Gomez-Ius R., Loza E., Navarro M., Olay T. (1983). International collaborative study on standardization of bacterial sensitivity to fosfomycin. J. Antimicrob. Chemother..

[13] Asempa T.E., Abdelraouf K., Nicolau D.P. (2020). Metallo-β-lactamase resistance in Enterobacteriaceae is an artefact of currently utilized antimicrobial susceptibility testing methods. J. Antimicrob. Chemother..

[14] Reller L.B., Weinstein M., Jorgensen J.H., Ferraro M.J. (2009). Antimicrobial susceptibility testing: A review of general principles and contemporary practices. Clin. Infect. Dis..

[15] Humphries R.M., Ambler J., Mitchell S.L., Castanheira M., Dingle T., Hindler J.A., Koeth L., Sei K. (2018). CLSI methods development and standardization working group best practices for evaluation of antimicrobial susceptibility tests. J. Clin. Microbiol..

[16] Schuurmans J.M., Hayali A.S.N., Koenders B.B., ter Kuile B.H. (2009). Variations in MIC value caused by differences in experimental protocol. J. Microbiol. Methods.

[17] Van Belkum A., Dunne W.M. (2013). Next-generation antimicrobial susceptibility testing. J. Clin. Microbiol..

[18] Puttaswamy S., Gupta S., Regunath H., Smith L., Sengupta S. (2018). A comprehensive review of the present and future antibiotic susceptibility testing (AST) systems. Arch. Clin. Microbiol..

[19] Ellington M., Ekelund O., Aarestrup F.M., Canton R., Doumith M., Giske C., Grundman H., Hasman H., Holden M., Hopkins K.L. (2017). The role of whole genome sequencing in antimicrobial susceptibility testing of bacteria: Report from the EUCAST Subcommittee. Clin. Microbiol. Infect..

[20] Su M., Satola S.W., Read T.D. (2019). Genome-based prediction of bacterial antibiotic resistance. J. Clin. Microbiol..

[21] Goldberg B., Sichtig H., Geyer C., Ledeboer N., Weinstock G.M. (2015). Making the leap from research laboratory to clinic: Challenges and opportunities for next-generation sequencing in infectious disease diagnostics. MBio.

[22] Ferreira I., Beisken S., Lueftinger L., Weinmaier T., Klein M., Bacher J., Patel R., von Haeseler A., Posch A.E. (2020). Species identification and antibiotic resistance prediction by analysis of whole-genome sequence data by use of ARESdb: An analysis of isolates from the Unyvero lower respiratory tract infection trial. J. Clin. Microbiol..

[23] Ersoy S.C., Abdelhady W., Li L., Chambers H.F., Xiong Y.Q., Bayer A.S. (2019). Bicarbonate resensitization of methicillin-resistant *Staphylococcus aureus* to β-Lactam antibiotics. Antimicrob. Agents Chemother..

[24] Kumaraswamy M., Lin L., Olson J., Sun C.-F., Nonejuie P., Corriden R., Döhrmann S., Ali S.R., Amaro D., Rohde M. (2016). Standard susceptibility testing overlooks potent azithromycin activity and cationic peptide synergy against MDR *Stenotrophomonas maltophilia*. J. Antimicrob. Chemother..

[25] Lin L., Nonejuie P., Munguia J., Hollands A., Olson J., Dam Q., Kumaraswamy M., Rivera H., Corriden R., Rohde M. (2015). Azithromycin synergizes with cationic antimicrobial peptides to exert bactericidal and therapeutic activity against highly multidrug-resistant Gram-negative bacterial pathogens. EBioMedicine.

[26] Ersoy S.C., Otmishi M., Milan V.T., Li L., Pak Y., Mediavilla J., Chen L., Kreiswirth B., Chambers H.F., Proctor R.A. (2020). Scope and predictive genetic/phenotypic signatures of ‘bicarbonate [NaHCO_3_]-responsiveness’ and β-Lactam sensitization among methicillin-resistant *Staphylococcus aureus* (MRSA). Antimicrob. Agents Chemother..

[27] Rose W.E., Bienvenida A.M., Xiong Y.Q., Chambers H.F., Bayer A.S., Ersoy S.C. (2019). Ability of bicarbonate supplementation to sensitize selected methicillin-resistant *Staphylococcus aureus* (MRSA) strains to β-Lactam antibiotics in an *ex vivo* simulated endocardial vegetation model. Antimicrob. Agents Chemother..

[28] Ersoy S.C., Chambers H.F., Proctor R.A., Rosato A.E., Mishra N.N., Xiong Y.Q., Bayer A.S. (2021). Impact of Bicarbonate on PBP2a Production, Maturation, and Functionality in Methicillin-Resistant *Staphylococcus aureus*. Antimicrob. Agents Chemother..

[29] Harrison E.M., Ba X., Coll F., Blane B., Restif O., Carvell H., Köser C.U., Jamrozy D., Reuter S., Lovering A. (2019). Genomic identification of cryptic susceptibility to penicillins and β-lactamase inhibitors in methicillin-resistant *Staphylococcus aureus*. Nat. Microbiol..

[30] Cockerill F.R. (2012). Performance Standards for Antimicrobial Susceptibility Testing: Twenty-Second Informational Supplement.

[31] Patel J., Cockerill F., Alder J., Bradford P., Eliopoulos G., Hardy D., Hindler J., Jenkins S., Lewis J., Miller L. (2014). Performance Standards for Antimicrobial Susceptibility Testing: Twenty-Fourth Informational Supplement.

[32] Dien Bard J., Hindler J.A., Gold H.S., Limbago B. (2014). Rationale for eliminating *Staphylococcus* breakpoints for β-lactam agents other than penicillin, oxacillin or cefoxitin, and ceftaroline. Clin. Infect. Dis..

[33] Cockerill F., Patel J., Alder J., Bradford P., Dudley M., Eliopoulos G., Hardy D., Hecht D., Hindler J., Powell M. (2013). Performance Standards for Antimicrobial Susceptibility Testing: Twenty-Third Informational Supplement.

[34] Bamgbola O. (2016). Review of vancomycin-induced renal toxicity: An update. Ther. Adv. Endocrinol. Metab..

[35] Abraham G., Finkelberg D., Spooner L.M. (2008). Daptomycin-induced acute renal and hepatic toxicity without rhabdomyolysis. Ann. Pharmacother..

[36] Gould I., Reilly J., Bunyan D., Walker A. (2010). Costs of healthcare-associated methicillin-resistant *Staphylococcus aureus* and its control. Clin. Microbiol. Infect..

[37] Croxatto A., Prod’hom G., Faverjon F., Rochais Y., Greub G. (2016). Laboratory automation in clinical bacteriology: What system to choose?. Clin. Microbiol. Infect..

[38] Matuschek E., Brown D.F., Kahlmeter G. (2014). Development of the EUCAST disk diffusion antimicrobial susceptibility testing method and its implementation in routine microbiology laboratories. Clin. Microbiol. Infect..

[39] Skov R.L., Pallesen L.V., Poulsen R.L., Espersen F. (1999). Evaluation of a new 3-h hybridization method for detecting the *mecA* gene in *Staphylococcus aureus* and comparison with existing genotypic and phenotypic susceptibilty testing methods. J. Antimicrob. Chemother..

[40] Hiramatsu K., Asada K., Suzuki E., Okonogi K., Yokota T. (1992). Molecular cloning and nucleotide sequence determination of the regulator region of *mecA* gene in methicillin-resistant *Staphylococcus aureus* (MRSA). FEBS Lett..

[41] Cunningham S.A., Chia N., Jeraldo P.R., Quest D.J., Johnson J.A., Boxrud D.J., Taylor A.J., Chen J., Jenkins G.D., Drucker T.M. (2017). Comparison of Whole-Genome Sequencing Methods for Analysis of Three Methicillin-Resistant *Staphylococcus aureus* Outbreaks. J. Clin. Microbiol..

[42] Madigan T., Cunningham S.A., Patel R., Greenwood-Quaintance K.E., Barth J.E., Sampathkumar P., Cole N.C., Kohner P.C., Colby C.E., Asay G.F. (2018). Whole-genome sequencing for methicillin-resistant *Staphylococcus aureus* (MRSA) outbreak investigation in a neonatal intensive care unit. Infect. Control Hosp. Epidemiol..

[43] Cho H.K., Yang J.N., Cunningham S.A., Greenwood-Quaintance K.E., Dalton M.L., Collura C.A., Fang J.L., Heinrich A.L., Huskins W.C., Patel R. (2020). Molecular epidemiology of methicillin-susceptible *Staphylococcus aureus* in infants in a neonatal intensive care unit. Infect. Control Hosp. Epidemiol..

[44] Cunningham S.A., Jeraldo P.R., Schuetz A.N., Heitman A.A., Patel R. (2020). *Staphylococcus aureus* whole genome sequence–based susceptibility and resistance prediction using a clinically amenable workflow. Diagn. Microbiol. Infect. Dis..

[45] Patel R. (2021). Personal communication.

[46] Fenn W.O. (1928). The carbon dioxide dissociation curve of nerve and muscle. Am. J. Physiol.-Leg. Content.

[47] Hudzicki J. (2009). Kirby-Bauer disk diffusion susceptibility test protocol. Am. Soc. Microbiol..

[48] Mathema B., Mediavilla J., Kreiswirth B.N. (2008). Sequence analysis of the variable number tandem repeat in *Staphylococcus aureus* protein A gene. Bacterial Pathogenesis.

[49] Mediavilla J.R., Chen L., Mathema B., Kreiswirth B.N. (2012). Global epidemiology of community-associated methicillin-resistant *Staphylococcus aureus* (CA-MRSA). Curr. Opin. Microbiol..

[50] Chen L., Mediavilla J.R., Oliveira D.C., Willey B.M., de Lencastre H., Kreiswirth B.N. (2009). Multiplex real-time PCR for rapid staphylococcal cassette chromosome *mec* typing. J. Clin. Microbiol..

[51] Lina G., Boutite F., Tristan A., Bes M., Etienne J., Vandenesch F. (2003). Bacterial competition for human nasal cavity colonization: Role of staphylococcal *agr* alleles. Appl. Environ. Microbiol..

[52] Hershberger E., Coyle E.A., Kaatz G.W., Zervos M.J., Rybak M.J. (2000). Comparison of a rabbit model of bacterial endocarditis and an in vitro infection model with simulated endocardial vegetations. Antimicrob. Agents Chemother..

[53] Rose W.E., Leonard S.N., Rybak M.J. (2008). Evaluation of daptomycin pharmacodynamics and resistance at various dosage regimens against *Staphylococcus aureus* isolates with reduced susceptibilities to daptomycin in an in vitro pharmacodynamic model with simulated endocardial vegetations. Antimicrob. Agents Chemother..

[54] Rose W.E., Leonard S.N., Sakoulas G., Kaatz G.W., Zervos M.J., Sheth A., Carpenter C.F., Rybak M.J. (2008). Daptomycin activity against *Staphylococcus aureus* following vancomycin exposure in an *in vitro* pharmacodynamic model with simulated endocardial vegetations. Antimicrob. Agents Chemother..

[55] Standiford H.C., Jordan M.C., Kirby W.M. (1970). Clinical pharmacology of carbenicillin compared with other penicillins. J. Infect. Dis..

[56] Rao S.N., Rhodes N.J., Lee B.J., Scheetz M.H., Hanson A.P., Segreti J., Crank C.W., Wang S.K. (2015). Treatment outcomes with cefazolin versus oxacillin for deep-seated methicillin-susceptible *Staphylococcus aureus* bloodstream infections. Antimicrob. Agents Chemother..

