# Supplementary material for: A Combined Phenotypic-Genotypic Predictive Algorithm for In Vitro Detection of Bicarbonate: β-Lactam Sensitization among Methicillin-Resistant Staphylococcus aureus (MRSA)
Source: Antibiotics (Basel). 2021 Sep 9;10(9):1089. doi: 10.3390/antibiotics10091089 (PMC8469475; doi:10.3390/antibiotics10091089)
Supplement: Supplementary file 1 [file antibiotics-10-01089-s001.zip › antibiotics-1337340-supplementary.pdf]

antibiotics

Article

A Combined Phenotypic-Genotypic Predictive Algorithm for In Vitro Detection of Bicarbonate:β-Lactam Sensitization Among Methicillin-Resistant *Staphylococcus aureus* (MRSA)

Selvi C. Ersoy [1], Warren E. Rose [2], Robin Patel [3], Richard A. Proctor [4], Henry F. Chambers [5], Ewan M. Harrison [6], Youngju Pak [7] and Arnold S. Bayer [7,*]

1 The Lundquist Institute, Torrance, CA 90502, USA; selvi.ersoy@lundquist.org
2 School of Pharmacy, University of Wisconsin-Madison, Madison, WI 53705, USA; warren.rose@wisc.edu
3 Mayo Clinic, Rochester, MN 55902, USA; patel.robin@mayo.edu
4 School of Medicine and Public Health, University of Wisconsin, Madison, WI 53706-152, USA; rap@wisc.edu
5 School of Medicine, University of California San Francisco, San Francisco, CA 94143, USA; Henry.Chambers@ucsf.edu
6 Wellcome Sanger Institute, Hinxton, UK; Department of Medicine, University of Cambridge, Cambridge, UK; Department of Public Health and Primary Care, University of Cambridge, Cambridge, UK; eh6@sanger.ac.uk
7 The Lundquist Institute, Torrance, CA 90502, USA; Geffen School of Medicine at the University of California Los Angeles, Los Angeles, CA 90025, USA; ypak@lundquist.org (Y.P.); abayer@lundquist.org (A.S.B.)
* Correspondence: abayer@lundquist.org, 1-310-222-6422

Citation: Ersoy, S.C.; Rose, W.E.; Patel, R.; Proctor, R.A.; Chambers, H.F.; Harrison, E.M.; Pak, Y.; Bayer, A.S. A Combined Phenotypic-Genotypic Predictive Algorithm for In Vitro Detection of Bicarbonate: β-Lactam Sensitization among Methicillin-Resistant *Staphylococcus aureus* (MRSA). *Antibiotics* **2021**, *10*, 1089. https://doi.org/10.3390/antibiotics10091089

Received: 27 July 2021
Accepted: 30 August 2021
Published: 9 September 2021

Publisher's Note: MDPI stays neutral with regard to jurisdictional claims in published maps and institutional affil-iations.

Copyright: © 2021 by the authors. Licensee MDPI, Basel, Switzerland. This article is an open access article distributed under the terms and conditions of the Creative Commons Attribution (CC BY) license (http://creativecommons.org/licenses/by/4.0/).

Supplementary Materials

Table S1. Phenotypic and genotypic characteristics of NaHCO3-responsive and non-responsive strains.

Responsive Strains (n = 15)																	
	**Disk Diffusion Zone Diameter (mm)**			**E-Test MIC**[A]								**Cefazolin MIC (μg/mL)**			**Oxacillin MIC (μg/mL)**		
**Strain**	**PEN**	**AMP**	**AMOX +CA**[B]	**PEN + CA**	Susceptible Phenotype[C]	*mecA* Genotype	Ridom *spa* type	*agr* type	CC type	SCC*mec* type	β-lactamase (±)	CA-MHB	CA-MHB 100 mM Tris	CA-MHB Tris + $NaHCO_3$ [D]	CA-MHB	CA-MHB 100 mM Tris	CA-MHB Tris + $NaHCO_3$ [D]
MRSA 11/11	8 (R)	9 (R)	16 (S)	0.5	Yes	susceptible 2	t008	agr I	8	IV	+	16	16	0.5	32	32	0.5
MW2	9 (R)	11 (R)	20 (S)	0.38	Yes	susceptible 2	t128	agr III	1	IV	+	4	8	1	32	64	2
BCVA 289	8 (R)	9 (R)	16 (S)	1	Yes	susceptible 2	t008	agr I	8	IV	+	8	16	2	32	64	1
PB 031-038	6 (R)	6 (R)	11 (R)	12	No	resistant 2	Unknown[E]	agr I	8	IV	+	128	64	2	128	64	2
PB 004-193	8 (R)	9 (R)	15 (S)	1.5	Yes	susceptible 2	t008	agr I	8	IV	+	8	32	2	64	128	16
PB 043-043	8 (R)	8 (R)	15 (S)	1	Yes	susceptible 2	t008	agr I	8	IV	+	8	16	2	8	16	1
PB 077-107	7 (R)	9 (R)	13 (R)	1	Yes	susceptible 2	t002	agr II	5	II	+	64	64	2	128	32	4
C48	8 (R)	8 (R)	18 (S)	0.38	Yes	susceptible 2	t008	agr I	8	IV	+	8	32	2	8	16	1
C42	8 (R)	8 (R)	15 (S)	4	No	susceptible 2	t008	agr I	8	IV	+	64	128	4	32	128	8
C13	9 (R)	8 (R)	15 (S)	0.5	Yes	susceptible 2	t008	agr I	8	IV	+	8	16	2	16	64	2
C32	13 (R)	11 (R)	18 (S)	0.25	Yes	susceptible 2	t002	agr II	5	IV	+	8	8	2	16	32	8
C30	10 (R)	10 (R)	19 (S)	0.5	Yes	susceptible 2	t008	agr I	8	IV	+	16	8	1	16	16	1
C24	8 (R)	9 (R)	16 (S)	1	Yes	susceptible 2	t008	agr I	8	IV	+	8	16	1	16	32	1

C38	8 (R)	8 (R)	14 (R)	1.5	Yes	susceptible 2	t008	agr I	8	IV	+	16	16	1	32	64	1
RB 300-087	12 (R)	11 (R)	18 (S)	0.5	Yes	susceptible 2	t2265	agr I	45	IV	+	4	8	1	16	16	2

Non-responsive Strains (n = 15)

	Disk Diffusion Zone Diameter (mm)			E-Test MIC[A]								Cefazolin MIC (µg/mL)			Oxacillin MIC (µg/mL)		
Strain	PEN	AMP	AMOX +CA[B]	PEN + CA	Susceptible Phenotype[C]	*mecA* Genotype	Ridom *spa* type	*agr* type	CC type	SCC-*mec* type	β-lactamase (±)	CA-MHB	CA-MHB 100 mM Tris	CA-MHB Tris + Na-HCO_3 [D]	CA-MHB	CA-MHB 100 mM Tris	CA-MHB Tris + Na-HCO_3 [D]
BMC1001	7 (R)	8 (R)	15 (S)	8	No	resistant 2	t064	agr I	8	IV	+	256	256	256	128	256	256
C5	10 (R)	9 (R)	26 (S)	0.38	Yes	resistant 2	t242	agr II	5	II	+	8	16	4	32	64	64
RB 067-227	7 (R)	8 (R)	18 (S)	0.125	Yes	susceptible 2	t128	agr III	1	IV	+	32	64	32	32	32	64
RB 010-016	NZ[D]	7 (R)	11 (R)	24	No	resistant 2	t002	agr II	5	II	+	256	512	256	256	512	512
PB 027-133	8 (R)	8 (R)	13 (R)	1	Yes	resistant 2	t002	agr II	5	II	+	128	256	128	256	256	256
PB 088-180	8 (R)	8 (R)	23 (S)	0.25	Yes	resistant 2	t002	agr II	5	II	+	8	16	4	32	64	64
RB 034-221	8 (R)	9 (R)	23 (S)	0.047	Yes	resistant 2	t002	agr II	5	II	+	16	32	8	8	32	16
C7	7 (R)	8 (R)	14 (R)	4	No	susceptible 2	t008	agr I	8	IV	+	64	128	64	128	128	128
C36	8 (R)	8 (R)	14 (R)	12	No	resistant 2	t002	agr II	5	II	+	256	512	512	512	1024	1024
C15	7 (R)	9 (R)	16 (S)	3	No	resistant 2	t064	agr I	8	IV	+	128	256	128	256	256	1024
PB 300-111	9 (R)	8 (R)	17 (S)	0.125	Yes	susceptible 2	t051	agr I	8	IV	+	16	64	32	8	16	32

PB 321-236	10 (R)	10 (R)	23 (S)	0.032	Yes	resistant 2	t003	agr II	5	II	+	4	8	4	32	128	128
C3	7 (R)	7 (R)	14 (R)	0.095	Yes	susceptible 2	t008	agr I	8	IV	+	128	256	64	128	256	256
PB 017-037	10 (R)	11 (R)	24 (S)	1.5	Yes	resistant 2	t002	agr II	5	II	+	4	16	8	32	32	32
RB 057-171	9 (R)	9 (R)	15 (S)	8	No	susceptible 2	t9878	agr III	1	IV	+	1	2	2	8	16	32

[A] E-Test MIC units are μg/mL; [B] Zone diameter (mm) for amoxicillin + clavulanate (AMOX + CA) disk diffusion assay, classified as Resistant = (R); Susceptible = (S) based on newly defined breakpoint of R ≤ 14 mm and S ≥ 15 mm; [C] Penicillin MIC ≤ 2 in the presence of clavulanic acid; [D] 44 mM NaHCO3 used where indicated; [E] Unknown repeat succession: r11r19r12r05r25.

Table S2. Summary of statistical analyses of various algorithms for identifying NaHCO3-responsive MRSA strains.

**Screening Criteria**	**Linkage to $NaHCO_3$-Responsivness[A]**	**Sensitivity [95% CI]**	**Specificity [95% CI]**
AMOX + CA susceptible; *mecA* "susceptible 2 genotype"; *spa* type t008 or t002	0.002	66.7% [38.4–88.2]	100% [78.2–100]
mecA "susceptible 2 genotype"	0.009	93.3% [68.1–99.8]	66.7% [38.4–88.2]
mecA "susceptible 2 genotype"; *spa* type t008 or t002	0.009	80% [51.9–95.7]	86.7% [59.5–98.3]
AMOX + CA susceptible; *spa* type t008 or t002	0.08	66.7% [38.4–88.2]	80% [51.9–95.7]
AMOX + CA susceptible; *mecA* "susceptible 2 genotype"	0.01	80% [51.9–95.7]	80% [51.9–95.7]
AMOX + CA susceptible only	0.88	80% [51.9–95.7]	33.3% [11.8–61.6]
AMOX + CA susceptible; CC8	0.17	60% [32.3, 83.7]	80% [51.9–95.7]
AMOX + CA susceptible; *mecA* "susceptible 2 genotype"; CC8 or CC5	0.009	66.7% [38.4–88.2]	93.3% [68.1–99.8]
AMOX + CA susceptible; *agr* I	0.08	66.7% [38.4–88.2]	80% [51.9–95.7]
AMOX + CA susceptible; SCC*mec* IV	0.08	80% [51.9–95.7]	66.7% [38.4–88.2]

[A] Linkage of criteria to NaHCO3-responsiveness as determined by Chi-squared analysis.

Table S3. mecA sequencing primers.

Primer	Sequence (5' to 3')	Purpose
mecA F0	ACCGAA-GAAGTCGTGTCAGA	PCR amplification
mecA R2	ACGTTGTAAC-CACCCCAAGA	PCR amplification
mecA F1	ATTAACGTGGAGACGAG-CACT	Sequencing
mecA F2	CGGTAACATTGATCG-CAACG	Sequencing
mecA R3	TCGTCAAC-GATTGTGACACG	Sequencing
mecA R4	CGAAGGTATCATCTT-GTACCCA	Sequencing
